# Know your patient: psychological drivers of decision making

**DOI:** 10.1186/2045-4015-1-37

**Published:** 2012-09-24

**Authors:** Linda Emanuel

**Affiliations:** 1 Buehler Center on Aging, Health & Society, Northwestern University Feinberg School of Medicine, 750 N. Lake Shore Drive, Suite 601, Chicago, IL, 60611, USA

## Abstract

This is a commentary on “Attitudes of legal guardians of ventilated ICU patients toward the process of decision making associated with invasive nonlife-saving procedures” by Michael Kuniavsky, Freda DeKeyser Ganz, David M Linton, and Sigal Sviri. Kuniavsky and colleagues report that decision-making for the seriously ill is difficult for the patients’ legal guardians, many of whom would be comfortable with doctors making the decisions. This commentary offers that accurate predictions about treatment choices may be derived by using assessments that characterize the key drivers of individual’s decision making, thus relieving some of decision makers' burdens. This approach could also usher in an era of assessing quality of care for the seriously ill by whether the care matches patient goals.

## 

Kuniavsky and colleagues have established from yet one more perspective that decision making for seriously ill patients is difficult in many ways. This study profiles two especially hard aspects: gathering clear and useable information, and optimally sharing the decision-making process; and it additionally profiles the difficult situation of legal guardians (LGs), many of whom are next of kin [1]. Most guardians are, by virtue of the situation and their role, emotionally overwhelmed and likely to be in a partially psychologically refractory state as a result [2]. Most are, by the nature of the population, being pressed to function in a decision-making area well beyond their expertise. This study establishes that many LGs would be comfortable having the decision made without their LG role, confirming other commentators’ view that some situations favor emphasis of a more paternalistic than autonomy-driven model of decision making [3].

A solution is available that has been relatively little discussed and studied but has strong potential. It seems that most people’s decision-making drivers can be identified by asking them about scenario-based treatment goals. Using one such scenario-based worksheet, in which components of the decisions are arrayed in three domains-prognosis, disability, and treatment burden – over 70% of people displayed a clear threshold at which their goals shifted from interventionist-oriented to comfort-oriented [4]. We have described the possibility of routinely identifying a person’s deep psychological decision-making drivers in the three domains, even characterizing them in a triple code, so that clinicians and family members alike can base decisions in situations of patient decisional-incapacity on these reliable features [5]. For instance, using Likert Scales of 5 units with 5 being most interventionist, a person inclined to save life of any state at all cost might have a threshold of ‘Prognosis 5, Disability 5, Burden 5’ which might be succinctly reported as: P5-D5-B5. By contrast, a person inclined to emphasize quality of life over longevity might have a characterization of P1-D1-B1. Or a person with a characterization of P1-D2-B5 would be someone inclined to try hard if the prognosis were good and if the likely disability were modest but otherwise to emphasize comfort. It is known that goals of care selected by patients predict specific treatment choices well [6]. The studies must still be done, but it is highly likely that characterization of the underlying drivers of individuals’ decisions would predict specific treatment choices even better.

If patients had the fundamental drivers of their decisions characterized in this kind of fashion and if the characterization was available to the team, decision making would be much better. A clinician team and LG working together would know that the first patient in the above example would want all relevant life supports, while the second would want only comfort care interventions, and the third would want invasive interventions only if the prognosis were good and the chance of disability low. Such characterizations could be arrived at in routine surveys that can take 10–15 minutes [7] and can be done on the patient’s own time [8], including on mobile devices that can be electronically shared with the health care facility [9], and then routinely included or updated as part of a patient’s intake assessment so that virtually all patients would have such a characterization as part of getting to know them.

The notion is that deep drivers of decision making can be characterized and probably provide the most accurate and feasible rendition of substituted judgment for the patient. It also provides a way of making efficient and less emotionally burdensome decisions. It is compatible with the LG having something to rely on in times of emotional overload and allows a bridging of the paternalistic and autonomy-driven models of decision making.

Such an approach has another advantage of great policy significance. If each person were efficiently and accurately characterized, the match between their goals and services provided could be readily assessed. As such, these efficiently assessed goodness-of-match outcomes could also define what the optimal use of resource expenditure near the end of life is. That is, the decisions whether costly and burdensome or comfort oriented, could be judged by whether they are patient-goals warranted. Goal-warranted measures would become a routine area in public health and health services research. Perhaps Israel will be the first country to pioneer such a model in which costs, whether high or low, are warranted by the fact that they honor the patient’s preferences.

## Author information

Linda L. Emanuel, MD, PhD, is the Buehler Professor of Geriatric Medicine and director of the Buehler Center on Aging, Health & Society at the Northwestern University Feinberg School of Medicine. She is the principal and co-founder of the Education and implementation of Palliative and End-of-life Care (EPEC) Program and of the Patient Safety Education Program (PSEP) Program.
